# Supplementary material for: LY6E mediates an evolutionarily conserved enhancement of virus infection by targeting a late entry step
Source: Nat Commun. 2018 Sep 6;9:3603. doi: 10.1038/s41467-018-06000-y (PMC6127192; doi:10.1038/s41467-018-06000-y)
Supplement: Supplementary file 1 — Supplementary Information [file 41467_2018_6000_MOESM1_ESM.pdf]

Supplementary Information

LY6E mediates an evolutionarily conserved enhancement of virus infection by targeting a late entry step

Mar, K. *et al*.

Supplementary Figure 1. LY6E-mediated enhancement is dependent on cell type.

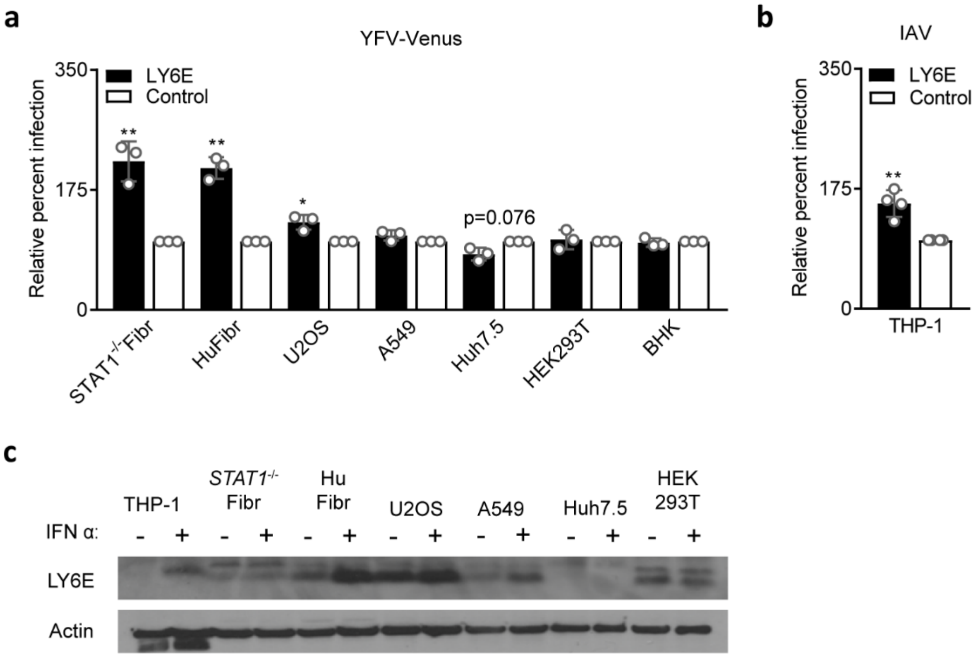

a LY6E or empty/fluc control constructs were expressed by lentivirus transduction in human *STAT1*$^{-/-}$ fibroblasts, immortalized wildtype human fibroblasts (HuFibr), U2OS, A549, Huh7.5, HEK293T, and BHK-21J cell lines. Cells were infected with YFV-17D-Venus (between 0.3 and 1 MOI, 24h). Percent infection was quantified by flow cytometry and is shown normalized to control. n = 3 biological replicates. **b** THP-1 transduced with lentivirus expressing LY6E or fluc control were infected with IAV (A/WSN/33, 0.02 MOI, 8h). Cells were permeabilized and stained for NP. Percent infection was quantified by flow cytometry and is shown normalized to fluc control. n = 4 biological replicates. **c** THP-1, *STAT1*$^{-/-}$ fibroblasts, HuFibr, U2OS, A549, Huh7.5 and HEK293T were mock treated or treated with 100 U/mL IFNα for 24 hours. Cells were lysed and probed for basal and IFN-induced LY6E as well as actin. * $p < 0.05$, ** $p < 0.01$. SD is shown. Data was analyzed prior to normalization by ratio paired t-test.

Supplementary Figure 2. LY6E knockdown reduces viral susceptibility.

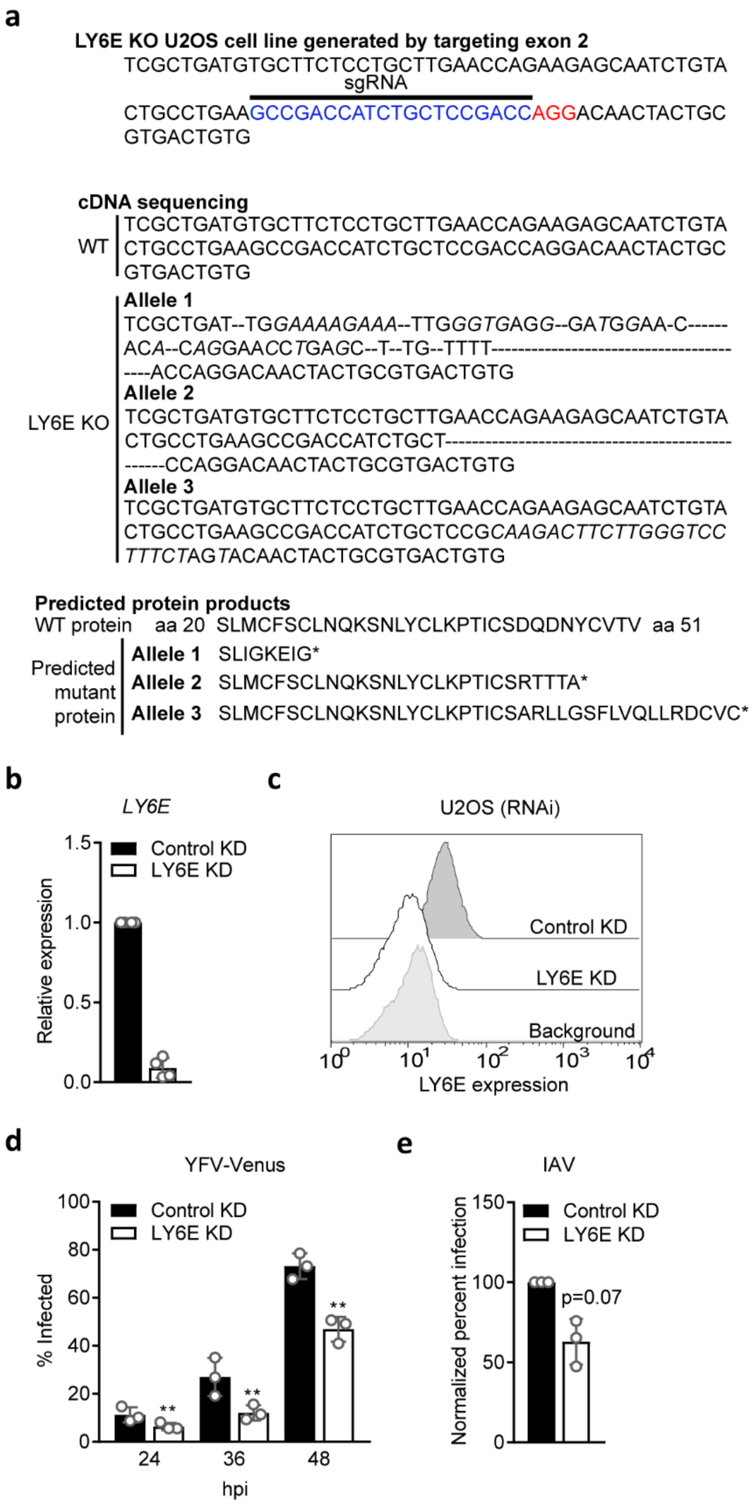

a Sequencing of clonal LY6E KO U2OS cell line. The sequence used to design a guide RNA to target exon 2 is indicated in blue. Red indicates the protospacer adjacent motif (PAM) site that is required for Cas9 nuclease targeting. cDNA sequencing all alleles of LY6E from triploid LY6E KO U2OS is shown. Predicted protein product from each mutated allele is also shown. Italicized letters indicate inserted nucleotides. Hyphens indicate deletions or gaps in the sequence relative to allele 3. **b** *LY6E* expression in U2OS that have been reverse transfected with LY6E-targeting or a non-silencing control (NSC) siRNA. Expression was determined by qRT-PCR using the delta-delta CT method, normalizing to LY6E expression in NSC cells. $n = 3$ biological replicates. **c** Cell surface levels of endogenous LY6E in U2OS reverse transfected with LY6E-targeting or NSC siRNA. Cells were stained with anti-LY6E antibody and analyzed by flow cytometry. Background levels were determined by staining with an isotype control. **d** U2OS reverse transfected with LY6E and NSC siRNA were infected with YFV-17D-Venus (0.7 MOI) and harvested for flow cytometry at 24, 36, and 48 hpi. Percent infection was quantified by flow cytometry. $n = 3$ biological replicates. **e** U2OS reverse transfected with LY6E and NSC siRNA were infected with IAV (A/WSN/33, 0.31 MOI, 8h). Cells were permeabilized and stained for NP. Percent infection was quantified by flow cytometry and is shown normalized to NSC-transfected cells. $n = 3$ biological replicates. ** $p < 0.01$. SD is shown. For **d** and **e**, ratio paired t-test was performed prior to normalization.

Supplementary Figure 3. LY6E does not affect the global transcriptome or IFN signaling.

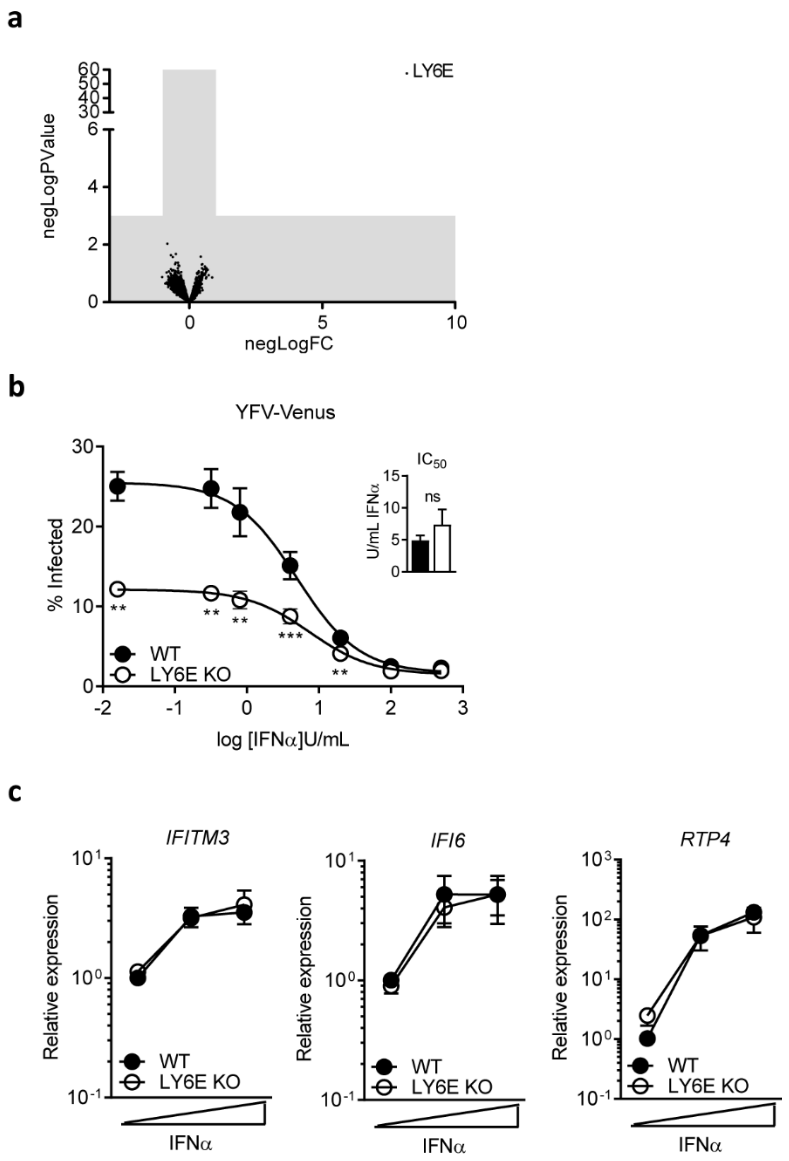

a RNA from STAT1$^{-/-}$ fibroblasts transduced with LY6E or empty vector control was isolated and submitted for RNA-Seq analysis. Log fold change (LogFC) is graphed against negative Log P value (negLogPValue). n = 3 biological replicates. **b** LY6E KO and WT U2OS were mock-treated or treated with 0.16 to 500 U/mL IFNα then infected with YFV-17D-Venus (0.43 MOI, 24h, N=3). Percent infection was quantified by flow cytometry. IC_{50} was calculated from nonlinear regression analysis using GraphPad Prism 5. LY6E KO R^2 = 0.9535. WT R^2 = 0.9668. P=0.2082. **c** LY6E KO and WT U2OS were treated for 4 hours with 0 to 500 U/mL of IFNα then lysed for qRT-PCR analysis of *IFITM3*, *IFI6*, and *RTP4* transcript induction. Expression was determined using the delta-delta Ct method, normalizing to expression of the respective genes in untreated WT U2OS. n = 3 biological replicates. ** $p < 0.01$, *** $p < 0.001$. SD is shown. Data was analyzed by ratio paired t-test.

Supplementary Figure 4. Phylogenetic analysis of LY6E orthologs.

a

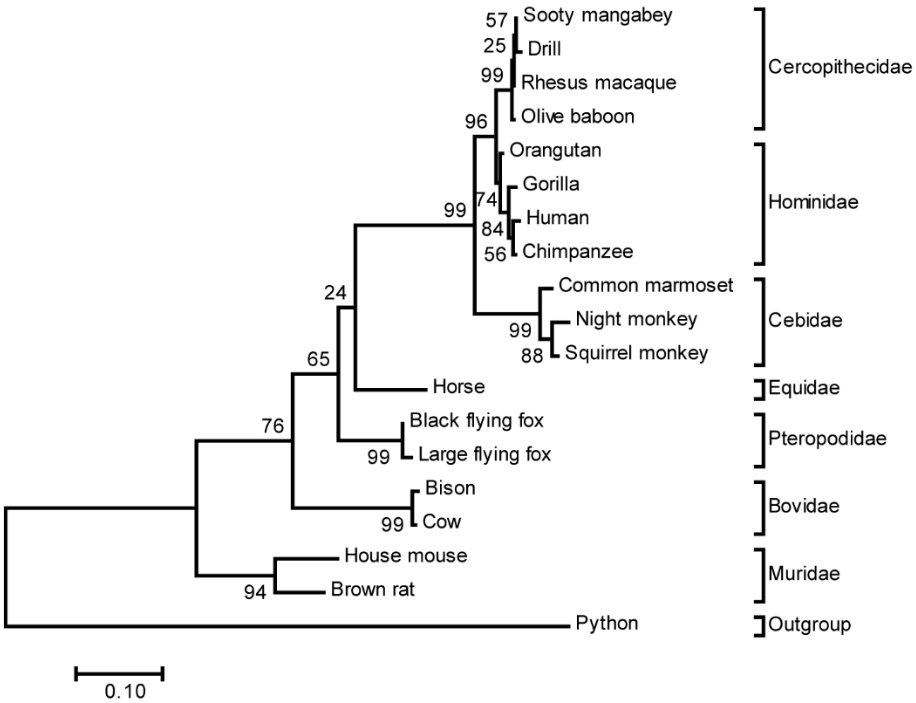

a Molecular phylogenetic analysis of LY6E ortholog nucleotide sequences by maximum likelihood method. Bootstrap values resulting from 1000 replicates are shown next to the branches. The tree is drawn to scale, with branch lengths measured in the number of substitutions per site. Evolutionary analyses were conducted in MEGA7.

Supplementary Figure 5. Site-directed mutagenesis of LY6E.

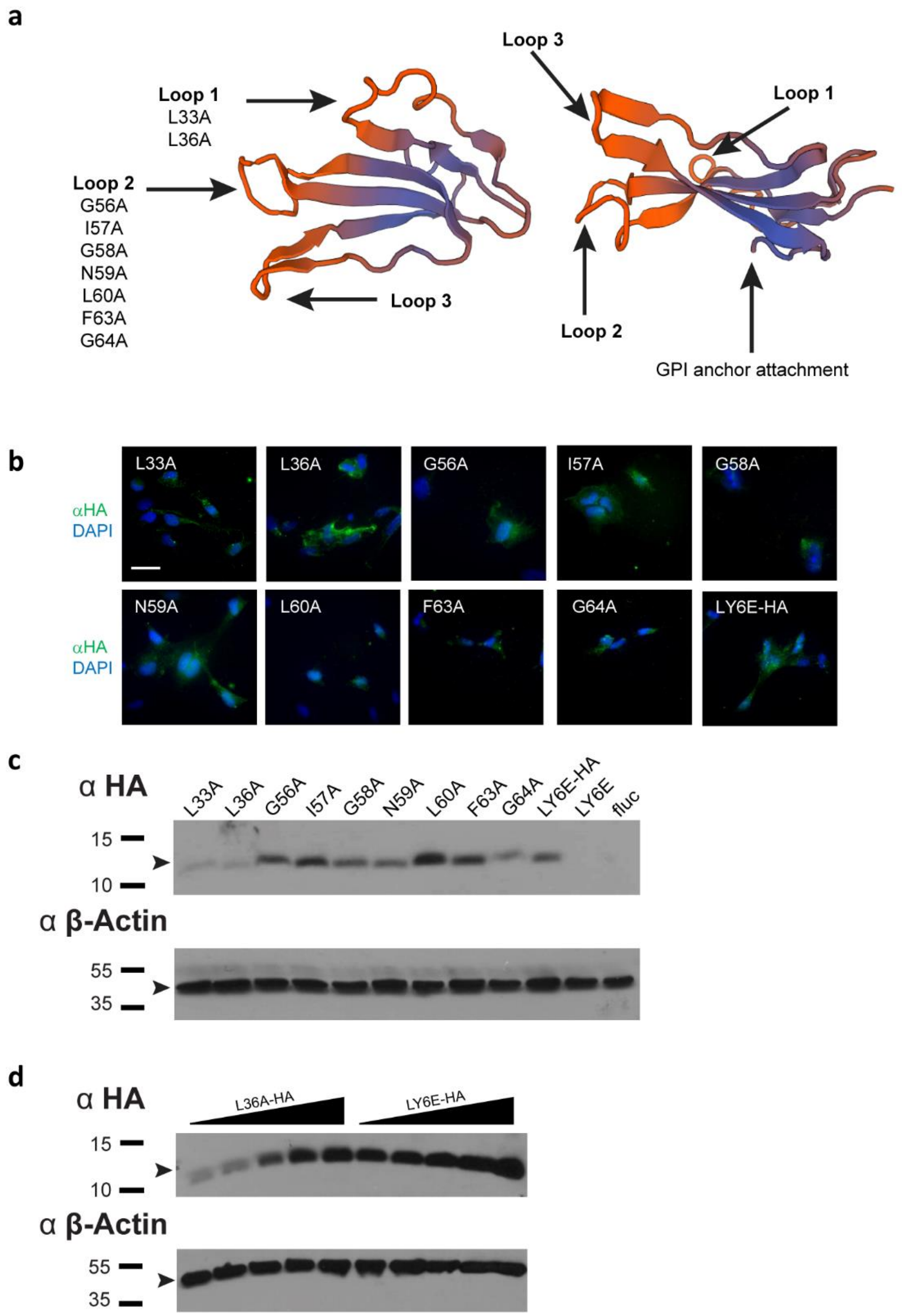

a Predicted structural model of LY6E rendered from a template of the structurally similar three finger protein irditoxin subunit B (2h7z.1.B, 25.35% shared sequence identity) by using SWISS-MODEL[1]. Orange indicates low quality modeling while blue indicates high quality modeling. The model was assigned a QMEAN Z-score of -2.13 which indicates that the model is comparable to what one would expect from experimental structures of similar size. LY6E point mutations are indicated at their respective loop regions. **b** *STAT1*$^{-/-}$ fibroblasts expressing LY6E-HA or HA-tagged point mutants shown in **a**. The cell surface was probed for HA (green) and DAPI was added to stain nuclei (blue). Scale bar, 20 µm. **c** Whole cell lysates from *STAT1*$^{-/-}$ fibroblasts described in **b** were lysed and probed for HA or actin expression. *n* = 1 of 3 biological replicates. **d** *STAT1*$^{-/-}$ fibroblasts were transduced with 5 volumes of lentivirus (two-fold increase per dose) expressing LY6E (L36A)-HA or LY6E-HA. Cells were lysed and whole cell lysate was probed for HA or actin expression. *n* = 1 of 3 biological replicates.

Supplementary Figure 6.

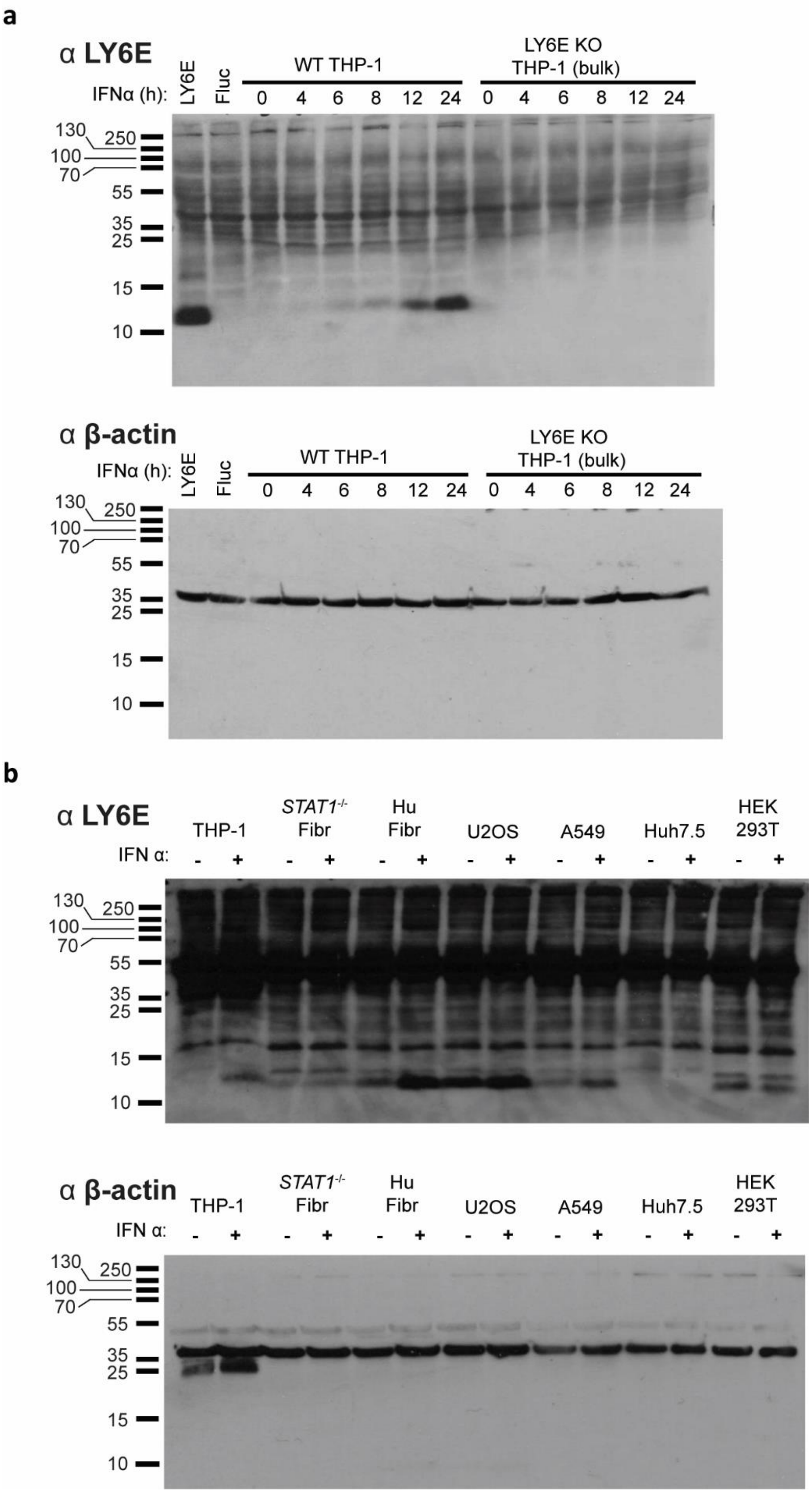

a Uncropped scan of blot shown in Figure 3a. **b** Uncropped scan of blot shown in Supplemental Figure 1c.

Supplementary Figure 7.

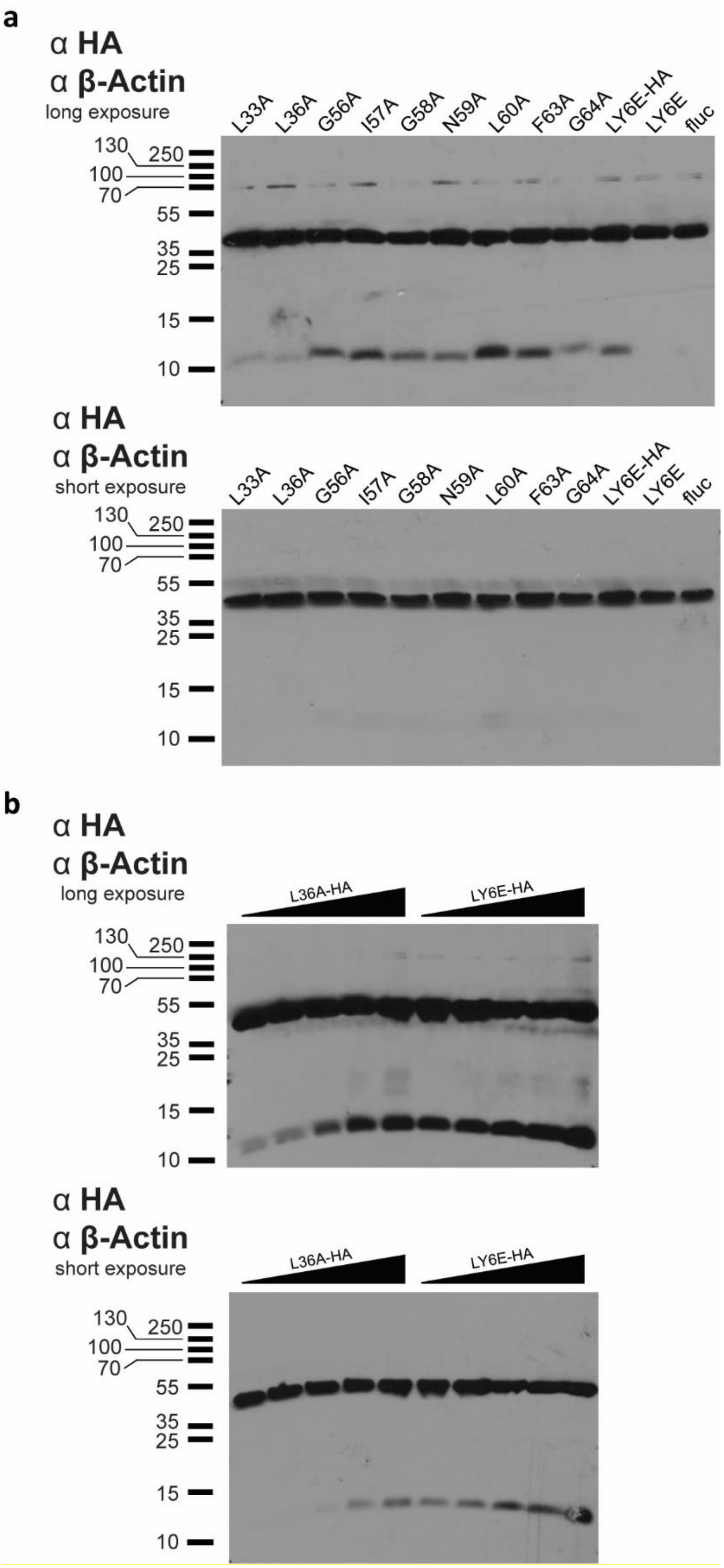

a Uncropped scan of blot shown in Supplemental Figure 5c. **b** Uncropped scan of blot shown in Supplemental Figure 5d.

Supplementary Figure 8.

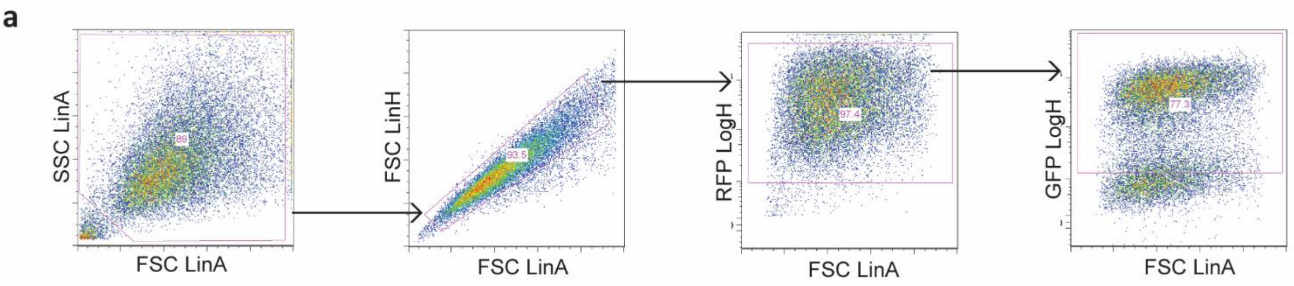

a General gating strategy for quantifying viral-infection (GFP-positive) in cells transduced with lentivirus co-expressing a gene of interest and TagRFP.

Supplementary Table 1. Phylogenetic analysis of LY6E orthologs.

LY6E Ortholog	Alignment Length	Identical Residues	Similar Residues	Percent identity	Percent similarity
Chimpanzee	131	131	0	100.00	100.00
Orangutan	131	130	1	99.24	100.00
Gorilla	131	130	1	99.24	100.00
**Rhesus macaque**	**131**	**127**	**3**	**96.95**	**99.24**
Baboon	131	127	3	96.95	99.24
Drill	131	127	3	96.95	99.24
Sooty mangabey	131	127	3	96.95	99.24
Squirrel monkey	131	111	11	84.73	93.13
Common marmoset	131	109	11	83.21	91.60
Night monkey	131	109	11	83.21	91.60
**Black flying fox**	**131**	**100**	**9**	**76.34**	**83.21**
Horse	131	100	10	76.34	83.97
Large flying fox	131	99	9	75.57	82.44
Cow	131	92	10	70.23	77.86
Bison	131	91	10	69.47	77.10
Brown Rat	132	82	15	62.12	73.48
**House mouse**	**132**	**80**	**17**	**60.61**	**73.48**
Python	131	43	20	32.82	48.09

Amino acid sequence identity and similarity of a subset of LY6E orthologs relative to human LY6E. The full sequence including the signal peptide and cleaved hydrophobic C-terminus was used for this analysis. The analysis was conducted using Sequence Manipulation Suite[2].

Supplementary Methods

Cloning

Overlap-extension PCR (OE PCR) was used to generate block and point mutations, as well as to add HA tags to LY6E and CD59. The published *P. alecto* Ly6e annotation was corrected by removing 99 amino acids prior to the predicted signal peptide, using OE PCR. BP arms were added to *M. musculus* Ly6e for Gateway cloning. All genes were transferred to Gateway-compatible pSCRPSY or pTRIP lentiviral backbones by LR reaction. sgRNA sequences for targeting LY6E were cloned into the lentiCRISPRv2 plasmid as detailed by the GeCKO protocol[3,4].

Primer name	Usage	Sequence
YFV-17D 5' NTR	qRT-PCR primer for YFV cold-bind assay	aatcgagttgctaggcaataaacac
YFV-17D capsid gene junction	qRT-PCR primer for YFV cold-bind assay	tccctgagctttacgaccaga
IAV A/WSN/33 HA forward	qRT-PCR primer for IAV cold-bind assay	taacctgctcgaagacagac
IAV A/WSN/33 HA reverse	qRT-PCR primer for IAV cold-bind assay	agagccatccggtgatgtta
Bat Ly6e repair forward	Repair *P. alecto* Ly6e cDNA	agacctgaattcggtaccatgaaggtctttctgcctgtgc
Bat Ly6e repair reverse	Repair *P. alecto* Ly6e cDNA	atataactcgagctaggggcccagtctgagg
Mouse Ly6e BP arms forward	Add BP arms to mouse Ly6e cDNA	ggggacaagtttgtacaaaaaagcaggcttcaccatgtctgccacttccaacatgagagt cttcctgc
Mouse Ly6e BP arms reverse	Add BP arms to mouse Ly6e cDNA	ggggaccactttgtacaagaaagctgggttcaggggctcagctgcagcagagccaaca agc
Human LY6E sgRNA #4 forward	Oligo to target LY6E exon 2	caccggccgaccatctgctccgacc
Human LY6E sgRNA #4 reverse	Oligo to target LY6E exon 2	aaacggtcggagcagatggtcggcc
Human LY6E surveyor nuclease forward	Amplify CRISPR-targeted region to confirm editing	ttcctcggaactgagtaacatcaggaatgg
Human LY6E surveyor nuclease reverse	Amplify CRISPR-targeted region to confirm editing	tgggggacatctgtatttatttgaggcacc
LY6E exon 2 genomic DNA forward	Amplify and sequence LY6E exon 2 after CRISPR	ggcctggccacactgtctcac
LY6E exon 2 genomic DNA reverse	Amplify and sequence LY6E exon 2 after CRISPR	ccaagggcacagatcaggcacg
pENTR backbone NspI forward	Backbone forward primer for OE PCR	ctggccttttgctggccttttgctcacatgttctttcctgcgttatcccc
pENTR backbone PvuI reverse	Backbone reverse primer for OE PCR	tcaaccaaaccgttattcattcgtgattgcgcctgagcgagacgaaatacgcgatcgctg

LY6E-HA tag forward	Add HA tag to LY6E before GPI anchor by OE PCR	actagcgtaatctggaacatcgtatgggtagaaattgcacagaaagctctggcagca
LY6E-HA tag reverse	Add HA tag to LY6E before GPI anchor by OE PCR	ttctacccatacgatgttccagattacgctagtgcggccgatggcgggct
CD59-HA tag forward	Add HA tag to CD59 before GPI anchor by OE PCR	acgatgttccagattacgctaatggtgggacatccttatcagag
CD59-HA tag reverse	Add HA tag to CD59 before GPI anchor by OE PCR	ctctgataaggatgtcccaccattagcgtaatctggaacatcgt
Block ASM forward 1	Mutate AA 21-24 to alanine	ttgctcttctggttcaagcaggaggcgcacgcagccgagctggctcgctccaca
Block ASM reverse 1	Mutate AA 21-24 to alanine	tgtggagcgagccagctcggctgcgtgcgcctcctgcttgaaccagaagagcaa
Block ASM forward 2	Mutate AA 25-28 to alanine (not cysteine)	aggcagtacagattgctcttctgggccgcgcaggcgaagcacatcagcgagctggct
Block ASM reverse 2	Mutate AA 25-28 to alanine (not cysteine)	agccagctcgctgatgtgcttcgcctgcgcggcccagaagagcaatctgtactgcct
Block ASM forward 3	Mutate AA 29-32 to alanine	ggcttcaggcagtacagattgctcgccgcggccgcgcaggagaagcacatcagcga
Block ASM reverse 3	Mutate AA 29-32 to alanine	tcgctgatgtgcttctcctgcgcggccgcggcgagcaatctgtactgcctgaagcc
Block ASM forward 4	Mutate AA 33-36 to alanine (not cysteine)	tcggagcagatggtcggcttcgcgcaggccgcattgctcttctggttcaagcagga
Block ASM reverse 4	Mutate AA 33-36 to alanine (not cysteine)	tcctgcttgaaccagaagagcaatgcggcctgcgcgaagccgaccatctgctccga
Block ASM forward 5	Mutate AA 37-40 to alanine	gtagttgtcctggtcggagcaggcggccgccgccaggcagtacagattgctcttct
Block ASM reverse 5	Mutate AA 37-40 to alanine	agaagagcaatctgtactgcctggcggcggccgcctgctccgaccaggacaactac
Block ASM forward 6	Mutate AA 41-44 to alanine (not cysteine)	gacacagtcacgcagtagttgtccgcggcggcgcagatggtcggcttcaggcagta
Block ASM reverse 6	Mutate AA 41-44 to alanine (not cysteine)	tactgcctgaagccgaccatctgcgccgccgcggacaactactgcgtgactgtgtc
Block ASM forward 7	Mutate AA 45-48 to alanine (not cysteine)	ggcactagcagacacagtcacgcaggcggcggcctggtcggagcagatggtcg
Block ASM reverse 7	Mutate AA 45-48 to alanine (not cysteine)	cgaccatctgctccgaccaggccgccgcctgcgtgactgtgtctgctagtgcc
Block ASM forward 8	Mutate AA 49-52 to alanine	attcccaatgccggcactagcagccgcagccgcgcagtagttgtcctggtcgga
Block ASM reverse 8	Mutate AA 49-52 to alanine	tccgaccaggacaactactgcgcggctgcggctgctagtgccggcattgggaat
Block ASM forward 9	Mutate AA 53-56 to alanine	gccaaatgtcacgagattcccaatggcggcagcagcagacacagtcacgcagtagttgt c
Block ASM reverse 9	Mutate AA 53-56 to alanine	gacaactactgcgtgactgtgtctgctgctgccgccattgggaatctcgtgacatttggc

Block ASM forward 10	Mutate AA 57-60 to alanine	caggctgtggccaaatgtcacggcagccgcagcgccggcactagcagacaca
Block ASM reverse 10	Mutate AA 57-60 to alanine	tgtgtctgctagtgccggcgctgcggctgccgtgacatttggccacagcctg
Block ASM forward 11	Mutate AA 61-64 to alanine	acaggtcttgctcaggctgtgggcagctgccgcgagattcccaatgccggcacta
Block ASM reverse 11	Mutate AA 61-64 to alanine	tagtgccggcattgggaatctcgcggcagctgcccacagcctgagcaagacctgt
Block ASM forward 12	Mutate AA 65-68 to alanine	aggccggggaacaggtcttggccgcggcggcgccaaatgtcacgagattcccaat
Block ASM reverse 12	Mutate AA 65-68 to alanine	attgggaatctcgtgacatttggcgccgccgcggccaagacctgttccccggcct
Block ASM forward 13	Mutate AA 69-72 to alanine (not cysteine)	ggatggggcaggccggggcacaggccgcgctcaggctgtggccaaatgtc
Block ASM reverse 13	Mutate AA 69-72 to alanine (not cysteine)	gacatttggccacagcctgagcgcggcctgtgccccggcctgccccatcc
Block ASM forward 14	Mutate AA 73-76 to alanine (not cysteine)	ccaacattgacgccttctgggatggcgcaggccgcggaacaggtcttgctcaggct
Block ASM reverse 14	Mutate AA 73-76 to alanine (not cysteine)	agcctgagcaagacctgttccgcggcctgcgccatcccagaaggcgtcaatgttgg
Block ASM forward 15	Mutate AA 77-80 to alanine	atggaagccacaccaacattgacggctgctgcggcggggcaggccggggaa
Block ASM reverse 15	Mutate AA 77-80 to alanine	ttccccggcctgccccgccgcagcagccgtcaatgttggtgtggcttccat
Block ASM forward 16	Mutate AA 81-84 to alanine	ctgatgcccatggaagccacagcagcagcggcgccttctgggatggggcag
Block ASM reverse 16	Mutate AA 81-84 to alanine	ctgccccatcccagaaggcgccgctgctgctgtggcttccatgggcatcag
Block ASM forward 17	Mutate AA 85-88 to alanine	tctggcagcagctgatgcccgcggcagccgcaccaacattgacgccttctgggat
Block ASM reverse 17	Mutate AA 85-88 to alanine	atcccagaaggcgtcaatgttggtgcggctgccgcgggcatcagctgctgccaga
Block ASM forward 18	Mutate 89-92 to alanine (not cysteine)	gaaattgcacagaaagctctggcagcaggcggcggccatggaagccacaccaacatt ga
Block ASM reverse 18	Mutate 89-92 to alanine (not cysteine)	tcaatgttggtgtggcttccatggccgccgcctgctgccagagctttctgtgcaatttc
Block ASM forward 19	Mutate 93-96 to alanine (not cysteine)	atcggccgcactgaaattgcacagagcggccgcgcagcagctgatgcccatggaag
Block ASM reverse 19	Mutate 93-96 to alanine (not cysteine)	cttccatgggcatcagctgctgcgcggccgctctgtgcaatttcagtgcggccgat
Block ASM forward 20	Mutate 97-100 to alanine (not cysteine)	agcccgccatcggccgcagcggcattgcacgcaaagctctggcagcagctgatg
Block ASM reverse 20	Mutate 97-100 to alanine (not cysteine)	catcagctgctgccagagctttgcgtgcaatgccgctgcggccgatggcgggct
Point ASM forward 1	Mutate L33 to alanine (L33A)	ggagcagatggtcggcttcaggcagtacgcattgctcttctggttcaagc

Point ASM reverse 1	Mutate L33 to alanine (L33A)	gcttgaaccagaagagcaatgcgtactgcctgaagccgaccatctgctcc
Point ASM forward 2	Mutate L36 to alanine (L36A)	gagcagatggtcggcttcgcgcagtacagattgctcttctgg
Point ASM reverse 2	Mutate L36 to alanine (L36A)	ccagaagagcaatctgtactgcgcgaagccgaccatctgctc
Point ASM forward 3	Mutate G56 to alanine (G56A)	tcacgagattcccaatggcggcactagcagacacagtcacgcagt
Point ASM reverse 3	Mutate G56 to alanine (G56A)	actgcgtgactgtgtctgctagtgccgccattgggaatctcgtga
Point ASM forward 4	Mutate I57 to alanine (I57A)	gccaaatgtcacgagattccccgcgccggcactagcagac
Point ASM reverse 4	Mutate I57 to alanine (I57A)	gtctgctagtgccggcgcggggaatctcgtgacatttggc
Point ASM forward 5	Mutate G58 to alanine (G58A)	gccaaatgtcacgagattcgcaatgccggcactagcagacacagtc
Point ASM reverse 5	Mutate G58 to alanine (G58A)	gactgtgtctgctagtgccggcattgcgaatctcgtgacatttggc
Point ASM forward 6	Mutate N59 to alanine (N59A)	gccaaatgtcacgagcgccccaatgccggcactagcagac
Point ASM reverse 6	Mutate N59 to alanine (N59A)	gtctgctagtgccggcattggggcgctcgtgacatttggc
Point ASM forward 7	Mutate L60 to alanine (L60A)	ctgtggccaaatgtcaccgcattcccaatgccggcactagcagac
Point ASM reverse 7	Mutate L60 to alanine (L60A)	gtctgctagtgccggcattgggaatgcggtgacatttggccacag
Point ASM forward 8	Mutate F63 to alanine (F63A)	ctcaggctgtggcccgctgtcacgagattcccaatgcc
Point ASM reverse 8	Mutate F63 to alanine (F63A)	ggcattgggaatctcgtgacagcgggccacagcctgag
Point ASM forward 9	Mutate G64 to alanine (G64A)	ggaacaggtcttgctcaggctgtgcgcaaatgtcacgagattccc
Point ASM reverse 9	Mutate G64 to alanine (G64A)	gggaatctcgtgacatttgcgcacagcctgagcaagacctgttcc

siRNA-mediated gene silencing

LY6E siRNA (Qiagen SI03019415) or Allstars Negative Control siRNA (Qiagen 1027280) at 5 nM were used for knockdown using HiPerfect Transfection Reagent (Qiagen) according to the manufacturer's reverse transfection protocol for 6 well plates. Cells were collected 48 h after transfection and replated for infection in 24 well plates at a density of 70,000 cells per well for YFV-Venus infection and 150,000 cells per well for IAV infection.

RNA isolation and qRT-PCR

All RNA isolations were performed per protocol using RNeasy Mini Kit (Qiagen) with on column DNase (Qiagen) digest. 40 ng total RNA was analyzed by one-step qRT-PCR using QuantiFast SYBR Green RT-PCR kit (Qiagen) which was run on (Applied Biosciences 7500 Fast Real-Time PCR System) with the following program: 10 min at 50°C, 5 min at 95°C, 35 x (10 sec at 95°C, 30 sec at 60°C), followed by melting curve analysis. qRT-PCR primers were purchased from Qiagen (*LY6E*: QT00087521, *IFITM3*: QT00049238, *RTP4*: QT00202188, *IFI6*: QT00244503, normalized to *RPS11*: QT00061516).

IFNα IC_{50} assay
LY6E KO U2OS and WT U2OS were plated in a 48 well plate at 32,000 cells per well. The next day, cells were pre-treated for 4 hours with various amounts of human IFNα (PBL Interferon Source 11100-1) at 0.016 U/mL to 500 U/mL) diluted in 10% FBS/0.1 mM NEAA/DMEM. After 4 hours, media was aspirated and cells were infected with YFV-17D-Venus for 1 hour at 37°C. Media was then aspirated and replaced with complete media. Infections were harvested 24 hours post-infection for FACS analysis.

mRNA-seq
RNA from uninfected LY6E and control *STAT1*$^{-/-}$ fibroblasts was isolated as described above and submitted to the UTSW McDermott Center Sequencing Core.

Samples were run on the Agilent 2100 Bioanalyzer to determine level of degradation thus ensuring only high-quality RNA is used (RIN Score 8 or higher). The Qubit fluorimeter was used to determine the concentration prior to staring library prep. Four micrograms of total DNAse-treated RNA were then prepared with the TruSeq Stranded Total RNA LT Sample Prep Kit from Illumina. Poly-A RNA was purified and fragmented before strand specific cDNA synthesis. cDNA were then a-tailed and indexed adapters were ligated. After adapter ligation, samples were PCR amplified and purified with AmpureXP beads, then validated again on the Agilent 2100 Bioanalyzer. Before being normalized and pooled, samples were quantified by Qubit then run on the Illumina Hiseq 2500 using SBS v3 reagents. Samples were subject to single-end, 50 bp read length whole transcriptome sequencing with 35 x 10^6 read coverage. The McDermott Center Bioinformatics Core used edgeR for differential expression and statistical analysis. Only genes that exceeded a logCPM of 1 were included for further statistical analysis using GraphPad Prism.

LY6E ortholog molecular phylogenetic analysis
LY6E sequences from nineteen species were aligned using MUSCLE[5] as implemented in MEGA7[6]. The alignment was manually curated to ensure preservation of coding sequences. A maximum likelihood phylogenetic tree was constructed by the HKY85 substitution model. The tree with the highest log likelihood (-2703.7050) is shown. Initial trees for the heuristic search were obtained automatically by applying Neighbor-Join and BioNJ algorithms to a matrix of pairwise distances estimated using the Maximum Composite Likelihood (MCL) approach, and then selecting the topology with superior log likelihood value.

The number of amino acid substitutions per site between sequences for the four indicated LY6E orthologs are shown. Analyses were conducted using the Dayhoff matrix-based model[7]. All positions with less than 95% site coverage were eliminated. That is, fewer than 5% alignment gaps, missing data, and ambiguous bases were allowed at any position. There were a total of 131 positions in the final dataset. Evolutionary analyses were conducted in MEGA7.

Structural prediction using SWISS-MODEL
The predicted structural model of LY6E was obtained using SWISS-MODEL[1].

Immunofluorescence
STAT1$^{-/-}$ fibroblasts transduced with lentivirus expressing LY6E point mutants were plated on poly-lysine coated chamber slides at 35,000 cells per well. Cells were fixed, stained with αHA epitope tag (16B12, 901501, BioLegend) for 30 min, then an AlexaFluor488-conjugated IgG for 30 min. Cells were stained with DAPI for 5 min.

Western blotting

STAT1$^{-/-}$ fibroblasts transduced with lentivirus expressing LY6E point mutants were lysed in RIPA buffer and sonicated. Lysates (5 µg) were run on a low molecular weight tricine gel. Protein was transferred to PVDF membrane, blocked with 5% milk/TBST, and probed for 1 hour at room temperature with 1:1000 αHA (16B12, 901501, BioLegend)/1:10,000 αβ-actin (ab6276, Abcam) diluted in 5% BSA/TBST. Membranes were washed 3 x 5 min in TBST then incubate for 30 min at room temperature with 1:2000 goat anti-mouse IgG conjugated to HRP and diluted in 5% milk/TBST. Membranes were washed again 3 x 5 min in TBST before ECL and preparation for film exposure and development. Uncropped blots are included in Supplementary Figures 6b and Supplementary 7a-b.

Supplementary References

1 Sanjana, N. E., Shalem, O. & Zhang, F. Improved vectors and genome-wide libraries for CRISPR screening. *Nat Methods* **11**, 783-784, doi:10.1038/nmeth.3047 (2014).

2 Shalem, O. *et al.* Genome-scale CRISPR-Cas9 knockout screening in human cells. *Science* **343**, 84-87, doi:10.1126/science.1247005 (2014).

3 Edgar, R. C. MUSCLE: multiple sequence alignment with high accuracy and high throughput. *Nucleic Acids Res* **32**, 1792-1797, doi:10.1093/nar/gkh340 (2004).

4 Kumar, S., Stecher, G. & Tamura, K. MEGA7: Molecular Evolutionary Genetics Analysis Version 7.0 for Bigger Datasets. *Mol Biol Evol* **33**, 1870-1874, doi:10.1093/molbev/msw054 (2016).

5 Schwartz, R. M. & Dayhoff, M. O. Protein and nucleic Acid sequence data and phylogeny. *Science* **205**, 1038-1039, doi:10.1126/science.205.4410.1038 (1979).

6 Biasini, M. *et al.* SWISS-MODEL: modelling protein tertiary and quaternary structure using evolutionary information. *Nucleic Acids Res* **42**, W252-258, doi:10.1093/nar/gku340 (2014).

7 Stothard, P. The sequence manipulation suite: JavaScript programs for analyzing and formatting protein and DNA sequences. *Biotechniques* **28**, 1102, 1104 (2000).
